# Enhanced STAT3 phosphorylation and PD-L1 expression in myeloid dendritic cells indicate impaired IL-27Ralpha signaling in type 1 diabetes

**DOI:** 10.1038/s41598-020-57507-8

**Published:** 2020-01-16

**Authors:** Z. Parackova, P. Vrabcova, I. Zentsova, J. Kayserova, I. Richtrova, L. Sojka, K. Stechova, Z. Sumnik, A. Sediva

**Affiliations:** 1 Department of Immunology, 2nd Faculty of Medicine, Charles University and Motol University Hospital, Prague, Czech Republic; 2 Sotio, A. S., Prague, Czech Republic; 3 Department of Internal Medicine, 2nd Faculty of Medicine, Charles University and Motol University Hospital, Prague, Czech Republic; 4 Department of Pediatrics, 2nd Faculty of Medicine, Charles University and Motol University Hospital, Prague, Czech Republic

**Keywords:** Cell signalling, Interleukins, Dendritic cells, Type 1 diabetes

## Abstract

Interleukin 27 (IL-27), a member of the IL-12 family, is important for T cell differentiation; however, little is known about its effect on dendritic cells (DCs). IL-27 can activate multiple signaling cascades, including the JAK/STAT pathway, and depending on the setting it can both promote and antagonize inflammatory responses. An anti-inflammatory function of IL-27 has been reported in several autoimmune diseases; however, in type 1 diabetes (T1D), an autoimmune disease where autoreactive cytotoxic T cells attack insulin-producing beta cells, IL-27 has been shown to have a dual role and contradictory effects. Here, we show impaired IL-27 signaling in a large cohort of T1D patients (n = 51) compared to age- and gender-matched healthy donors. Increased expression of the IL-27 receptor subunit *IL-27Ralpha* mRNA in purified myeloid DCs (mDCs), detected by gene expression microarrays was mirrored by enhanced signal transduction in T1D mDCs in response to IL-27 stimulation. Higher STAT phosphorylation in T1D patients was also accompanied by elevated expression of the inhibitory molecules *PD-L1*, *PD-L2* and *PD-1*, which may suggest not only immunomodulatory mechanisms of IL-27 in T1D but also a compensatory effort of T1D dendritic cells against the ongoing inflammation.

## Introduction

Type 1 diabetes (T1D) is a type 1 helper T cell-mediated autoimmune disease caused by the destruction of beta cells in the Langerhans islets of the pancreas. However, the involvement in disease development and progression is not limited to autoreactive T cells; in fact, a wide variety of immune cell populations producing different cytokines accompany the inflammatory response (insulitis) within the islets. Cytokines play pivotal roles in immune system activation and have been extensively studied in autoimmune diseases, including T1D^1–3^.

Interleukin 27 (IL-27) is a heterodimeric member of the IL-12 family, is composed of two subunits, p28 and EBV-induced gene 3 (EBI3) and is produced primarily by activated antigen-presenting cells^4,5^. The role of IL-27 is enigmatic because it can induce both pro- and anti-inflammatory responses. Although the first animal studies described IL-27 as an initiator of the Th1 response^5,6^, it was later found that IL-27 has broad inhibitory effects on Th1, Th2, and Th17 subsets and can induce IL-10-producing regulatory T cells (Tr1) in both mice and humans^7–9^.

IL-27 signals through a heterodimeric receptor composed of two subunits, gp130 and IL-27Ralpha (formerly called TCCR or WSX-1), which activate the STAT1 and STAT3 signaling pathways^10^. IL-27Ralpha exists in both a membrane and soluble form. Soluble IL-27Ralpha inhibits IL-27 binding to the cell surface and inhibits IL-27 signaling^11^.

IL-27 is known to act directly on T cells, suppressing their differentiation to effector T cells. Less is known about the effect of IL-27 on dendritic cells (DCs)^9,12,13^. IL-27 signaling in murine DCs limits pathogenic T cell responses, diminishes NLRP3 inflammasome activation and limits the ability of DCs to induce Th1 and Th17 differentiation and suppress autoimmune processes^14^.

The role of IL-27 in various autoimmune diseases has been reported^15^. IL-27 activates multiple signaling cascades and has both anti- and proinflammatory activities in different autoimmune diseases. However, with some exceptions, the anti-inflammatory effect of IL-27 has been mostly observed^15^.

In T1D, the role of IL-27 has been insufficiently investigated, and the studies are conflicting. Wang *et al*. showed that IL-27 has a pathogenic role in T cell-mediated autoimmune diabetes, using (nonobese diabetic) NOD mice^16^. On the other hand, Fujimoto *et al*. reported that mice lacking IL-27 or IL-27 receptor subunit had increased blood sugar levels, islet infiltration and proinflammatory cytokine levels in a streptozotocin-induced diabetes model^17^, suggesting that IL-27 could represent a potential target for therapeutic strategy. In humans, newly diagnosed T1D patients have increased IL-27 levels in the peripheral blood, and those levels strongly correlate with circulating Th17 cytokines, supporting the hypothesis that IL-27 can have a regulatory function^3^. An association between IL-27 polymorphisms and T1D was also reported in one genome-wide association study^18^, but this association was not confirmed in another report^19^.

In this study, we report for the first time the increased expression of IL-27Ralpha in myeloid dendritic cells (mDCs) in T1D patients, resulting in higher STAT3 phosphorylation and *PD-L1* expression in mDCs, suggesting a substantial immunomodulatory role in T1D pathogenesis.

## Results

### Increased IL-27Ralpha expression in myeloid dendritic cells in T1D patients

**Figure 1:**
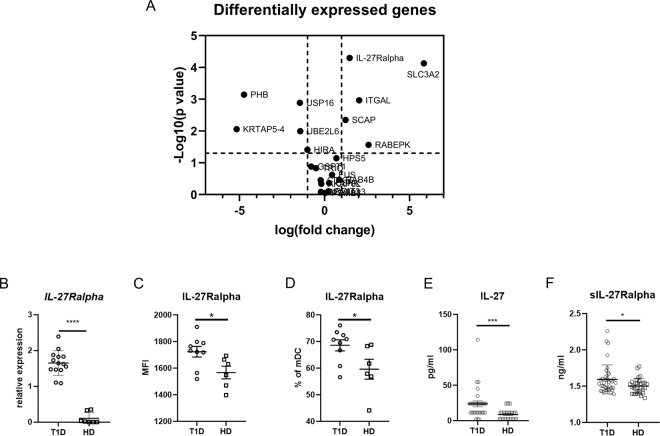
*IL-27Ralpha* expression. (**A**) Volcano plot of differentially expressed genes in T1D patients compared to healthy controls. Analysis of gene arrays was performed on sorted myeloid dendritic cells (mDCs) from T1D patients (n = 20) and healthy controls (n = 10). (**B**) *IL-27Ralpha* expression validation by RT PCR normalized to *GADPH*. Analysis was performed on sorted mDCs from 14 T1D patients and 7 healthy donors (HD). Surface expression of IL-27Ralpha on mDC expressed as (**C**) MFI and **(D)** percentage of IL-27Ralpha + CD11c + mDCs detected by flow cytometry in 9 T1D patients and 6 HD. (**E**) Serum levels of IL-27 in 30 T1D patients and 23 healthy donors detected by Luminex. (**F**) Serum levels of soluble IL-27Ralpha in T1D patients (n = 40) and healthy donors (n = 32) detected by ELISA. Statistical analysis was performed using the Mann-Whitney unpaired *t*-test. Values of p < 0.05 (*), p < 0.01 (**), p < 0.001 (***) and p < 0.0001 (****) were considered statistically significant.

By performing gene array analysis, we discovered that the myeloid dendritic cells (mDCs) of T1D patients (n = 20) expressed significantly more *IL-27Ralpha* compared to healthy donors (n = 10) (Fig. 1A). This finding was confirmed by real-time PCR analysis, whereby increased levels of *IL-27Ralpha* were detected in purified patient mDCs compared to healthy donor mDCs (Fig. 1B). Flow cytometry verified the results on the protein level. Not only is the percentage of mDCs expressing IL-27Ralpha higher in T1D patients, but also the receptor subunit expression on the cells is elevated (Fig. 1C,D). No difference in the expression of the second subunit of IL-27 receptor, gp130, was found (Suppl. Fig. 1A). Notably, we have also examined the expression of metalloproteinase ADAM17, which regulates IL-27Ralpha subunit shedding from the cell surface and found lower expression on T1D mDC’s surface (Suppl. Fig. 1B), thus elucidating the enhanced IL-27Ralpha expression on mDCs. Next, we established, that the higher IL-27Ralpha levels were mirrored by IL-27 levels and were able to detect elevated levels of IL-27 in T1D patients’ serum (Fig. 1E). Moreover, analysis of soluble IL-27Ralpha levels in patient (n = 40) and healthy (n = 32) serum revealed that T1D patients also had increased levels of the soluble form receptor in their periphery compared to the controls (Fig. 1F). Even though, the source of sIL-27Ralpha in serum is indistinguishable, we noticed that T1D T cells expressed significantly lower levels of IL-27Ralpha on their surface in comparison to healthy donors (Suppl. Fig. 1C). Intense shedding of the IL-27Ralpha from T1D T cells might be partially responsible for the increased serum levels of sIL-27Ralpha, however the ADAM17 expression on T cells remain on the control range (data not shown).

### Elevated STAT1 and 3 phosphorylation and expression of STAT1- and 3-induced genes

**Figure 2:**
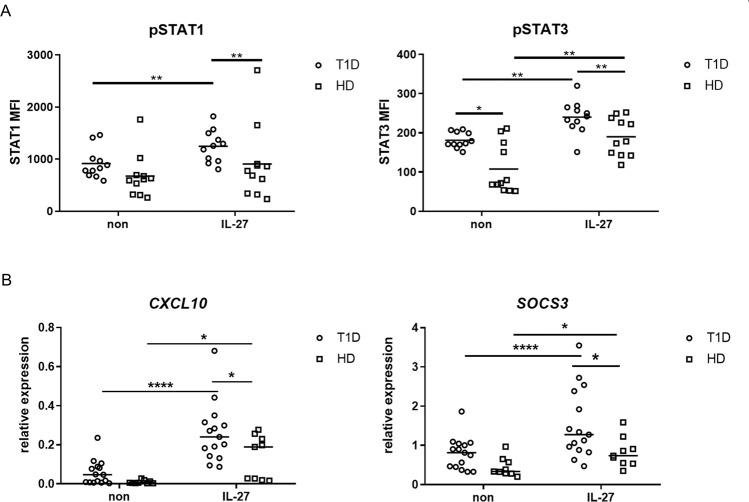
IL-27 signaling. (**A**) STAT1 (Tyr701) and STAT3 (Tyr705) phosphorylation in myeloid dendritic cells (mDCs) was analyzed by phosphoflow after whole blood from T1D patients (n = 11), and healthy donors (HD, n = 11) was stimulated with 100 ng/ml rhIL-27 for 15 minutes. Values are expressed as MFI (mean fluorescence intensity). (**B**) Expression of STAT1-induced *CXCL10* and STAT3-induced *SOCS3* after rhIL-27 (100 ng/ml) exposure in T1D patients (n = 16) and healthy donors PBMCs (n = 9) was normalized to *GADPH* and determined by RT PCR. Statistical analysis was performed using the Kruskal-Wallis test with Dunn’s multiple comparisons, Wilcoxon paired or Mann-Whitney unpaired *t*-test. Values of p < 0.05 (*), p < 0.01 (**), p < 0.001 (***) and p < 0.0001 (****) were considered statistically significant.

To uncover whether increased IL-27Ralpha expression has an impact on cell signaling, we analyzed STAT1 and STAT3 phosphorylation in mDCs of T1D patients (n = 11) and healthy donors (n = 11) using phosphoflow. We also analyzed pSTAT1 and pSTAT3 in plasmacytoid DCs (pDCs) as a control. The gating strategy is shown in Suppl. Fig. 1D. Upon rhIL-27 exposure, the STAT1 and STAT3 proteins were phosphorylated in DCs within 5 minutes and phosphorylation continued to rise with the maximum level reached in 15 minutes (Suppl. Fig. 2A). While analyzing the differences between patients and controls in the reactivity to rhIL-27 stimulation, we observed more profound STAT1 and STAT3 phosphorylation in patient mDCs compared to healthy control mDCs. Moreover, we also noticed slightly higher basal STAT3 phosphorylation in T1D mDCs (Fig. 2A). In pDCs, we did not detect any significant effect of rhIL-27 stimulation (data not shown) in comparison to controls. The effect of rhIL-27 stimulation on the PBMC compartment as detected by western blot is shown in Suppl. Fig. 2B and 3. To exclude impaired Janus kinases (JAK) activity as an underlying cause of the increased STAT3 phosphorylation, we stimulated T1D and control samples with rhIL-6 or rhIFNα to examine STAT3 and STAT1 phosphorylation, respectively. Both groups reacted to the cytokine exposure, however no differences between T1D and HD were detected (Suppl. Fig. 2C), suggesting that the detected increase of STAT3 activity in T1D mDCs is directly due to IL-27 stimulation and not due to a general JAK hyperactivation in T1D patients.

To confirm the effect of rhIL-27 on the signal transduction through STAT1 and STAT3 molecules, we analyzed the expression of STAT1- and STAT3-induced genes *CXCL10* and *SOCS3*, respectively, in the PBMCs of T1D patients (n = 16) and healthy donors (n = 9) upon rhIL-27 stimulation. Both groups were able to upregulate the expression of the genes; however, in T1D patients, the increase was more significant (Fig. 2B). To exclude the possible role of poor control of glucose metabolism in T1D patients in observed impaired IL-27 signaling, we correlated glycated hemoglobin (HbA1c) levels with STAT1, 3 phosphorylation, *CXCL10* and *SOCS3* expression. We have not found any significant relationship between glucose metabolism and IL-27 signaling (data not shown). Moreover, we also performed similar set of experiments with rhIL-27 stimulation on T2D samples to confirm that impaired IL-27 reactivity of T1D mDCs is not due to poor glucose control. As we did not notice any noteworthy differences between T2D patients and healthy donors, suggesting that observed increased STAT phosphorylation in patients’ mDCs is T1D specific (Suppl. Fig. 4B).

### IL-27 induces PD-L1 expression

**Figure 3:**
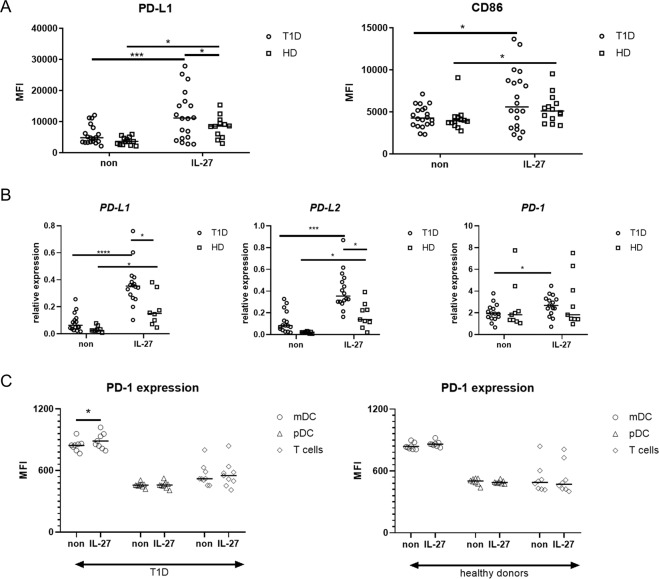
PD-L1 and CD86 expression on the cell surface. (**A**) After overnight incubation of PBMCs from T1D patients (n = 20) and healthy donors (n = 14) with rhIL-27 (100 ng/ml), the expression of PD-L1 and CD86 on the surface of mDCs was analyzed by flow cytometry. (**B)** The expression of *PD-L1, PD-L2* and *PD-1* normalized to GADPH was obtained by RT PCR in PBMCs from T1D patients (n = 16) and healthy donors (HD, n = 9) after stimulation with rhIL-27 (100 ng/ml) for 5 hours. (**C**) PD-1 expression on surface of mDC, pDC and T cells was analyzed by flow cytometry in T1D patients (n = 9) and HD (n = 8) after overnight incubation with 100 ng/ml rhIL-27 or left untreated. Statistical analysis was performed using the Kruskal-Wallis test with Dunn’s multiple comparisons the Wilcoxon paired or Mann-Whitney unpaired *t*-test. Values of p < 0.05 (*), p < 0.01 (**), p < 0.001 (***) and p < 0.0001 (****) were considered statistically significant.

Using flow cytometry, we detected the expression of PD-L1 and CD86 after rhIL-27 exposure on the surface of mDCs (Fig. 3A) from T1D patients (n = 19) and healthy controls (n = 14). Both T1D patients and healthy donors (HD) were able to significantly upregulate the expression of PD-L1 on the surface of mDCs in response to rhIL-27 stimulation; however the raise on T1D mDCs was more profound (Fig. 3A). There was no significant difference in PD-L1 on pDC’s surface (data not shown) between healthy donors and T1D patients upon rhIL-27 exposure. Moreover, we also analyzed CD86 expression on both populations of DCs and observed that even though rhIL-27 increases CD86 expression, there were no statistically significant differences between T1D and HD (Fig. 3A). Similarly to STATs phosphorylation, we did not find any relationship between HbA1c levels and PD-L1 nor CD86 expression on mDCs. Phenotype changes of T2D patients in response to rhIL-27 stimulation were within the control range (Suppl. Fig. 4C).

Next, we analyzed the expression of programmed death-1 (PD) family members, *PD-L1*, *PD-L2* and *PD-1* upon rhIL-27 exposure in the PBMC compartment (Fig. 3B) by real-time PCR. *PD-L1* and *PD-L2* were significantly upregulated after rhIL-27 stimulation in both T1D patients (n = 16) and healthy controls (n = 9), but the IL-27-induced expression was significantly higher in T1D patients. Moreover, *PD-1* expression was induced by IL-27 only in T1D patients and not in healthy donors (Fig. 3B,C). The expression of *PD-1* negatively correlated with HbAc1 levels in both unstimulated as well as rhIL-27 stimulated state, but no relationship was found between metabolic control and *PD-L1* and *PD-L2* expression (data not shown).

Since PD-1 is also expressed on T cells as an activation marker, we executed additional analysis of rhIL-27 effect on PD-1 expression on various cell populations – mDC, pDC and T cells by flow cytometry and found that only T1D mDCs are able to upregulate PD-1 expression on their surface as an effect of rhIL-27 stimulation (Fig. 3C).

## Discussion

IL-27 is a potent immunoregulator that has drawn interest as a target for immunotherapy^15^. Therefore, understanding its activity and biological role under physiological and disease conditions is essential. In this study, we show enhanced expression and signaling of IL-27Ralpha in myeloid dendritic cells (mDCs) of patients with type 1 diabetes (T1D). Increased STAT1 and STAT3 phosphorylation was accompanied by an increased expression of PD-L1 on the surface of mDCs. Interestingly, we also observed elevated expression of inhibitory receptors in T1D PBMCs in response to IL-27 stimulation, potentially suggesting a role for IL-27Ralpha signaling in the tolerogenic compensatory control of T1D.

IL-27 has been implicated in the pathologies of several autoimmune diseases, such as multiple sclerosis, systemic lupus erythematosus, rheumatoid arthritis, autoimmune gastritis and inflammatory bowel disease^15^. The exact mechanisms by which IL-27 contributes to the pathogenesis of these autoimmune diseases require further investigation because results from experimental models of autoimmune diseases have shown contradictory and not fully defined dual roles of IL-27^20^. Both pro- and anti-inflammatory activities have been reported, and the effect of IL-27 was often context-dependent, including in the studies focused on T1D. In NOD mice, IL-27 was reported to have a pathogenic role^16^, but in contrast, another report showed that mice lacking IL-27 signaling displayed diabetic symptoms, such as increased blood sugar levels, islet infiltration, proinflammatory cytokine production, and administration of IL-27 reversed these symptoms^17^, suggesting that targeting the IL-27 pathway could be a potential therapeutic strategy. One study performed in humans reported increased IL-27 levels in the peripheral blood of newly diagnosed T1D patients^3^, which is in agreement with our observation of elevated IL-27 level in our cohort of long-term treated T1D patients. Interestingly, we have previously identified IL-27 signaling as one of the pathways distinguishing two siblings who had already developed T1D out of four HLA identical quadruplets^21^. Elevated levels of IL-27 mRNA were also found in patients with active Crohn’s disease and an enhanced percentage of IL-27 immunoreactive cells in the gut mucosa in patients with active ulcerative colitis. However, the authors of the study suggested that enhanced IL-27 expression had an anti-inflammatory role in inflammatory bowel diseases due to the compensatory mechanism of the inflammatory process in the intestine^22^.

Increased IL-27 serum level was mirrored by elevated amount of the natural antagonist of IL-27, a soluble form of IL-27Ralpha, in long-term treated T1D patients. Soluble IL-27Ralpha (sIL-27Ralpha) has the ability to bind recombinant human IL-27, inhibit its binding to surface receptors and consequently block its STAT-mediated signaling^11^. Elevated levels of sIL-27Ralpha were also found in the sera of patients with Crohn’s disease, suggesting that this molecule may play a role in immunopathologies beyond T1D^11^.

Through gene microarrays, we detected increased expression of *IL-27Ralpha* in purified mDCs from T1D patients. IL-27Ralpha is constitutively expressed by numerous immune and nonimmune cell types^11^, and upon IL-27 stimulation, it activates the JAK/STAT and MAPK signaling pathways^10^. In this study, we observed elevated STAT1 and STAT3 phosphorylation after IL-27 exposure in T1D mDCs. Elevated STAT3 phosphorylation was also detected in unstimulated cells. STAT3 signaling has been shown to induce a tolerogenic phenotype in DCs, and conversely, STAT3 deficiency leads to the development of inflammation^23^. This might again suggest a tolerogenic role of IL-27 as a STAT3 activator in DCs. Moreover, IL-27 is a known inducer of *PD-L1* expression in various cell types, acting through STAT1 activation^24–26^, further implying an anti-inflammatory role for IL-27. Indeed, *in vitro* stimulation of T1D cells with IL-27 led not only to increased expression of *PD-L1* but also of *PD-L2*, and the expression was even further increased in T1D patients. Surprisingly, *PD-1* expression was also induced by IL-27; however, this effect was exclusive to mDCs of T1D patients. These inhibitory receptors are essential for regulating T cell activation and promoting immune tolerance. The PD-1/PD-L1 pathway regulates the induction and maintenance of peripheral immune tolerance and protects tissues against autoimmune attacks^27^. Mounting evidence has shown that impaired function of PD-1/PD-L1 signaling plays an important role in several autoimmune diseases, including T1D^28–31^.

Previous studies have associated IL-27 effect on regulatory T lymphocytes (Tregs) with both pro- and anti-inflammatory roles. The IL-27 signaling might contribute to their suppressive activities via STAT1-dependent manner^32,33^ or by attenuating the Th17-mediated inflammation as shown in an autoimmune model of experimental autoimmune encephalomyelitis^12^. On the other hand, Bin Dhuban *et al*. showed that IL-27 impairs the suppressive capacity of human Treg via the gp130 receptor subunit^34^. Even though we did not focus on Treg reactivity to IL-27 in our experimental setup, it is therefore plausible that Treg contribution to T1D pathology might be influenced by enhanced IL-27 signaling in T1D patients, however the exact spectrum of effects is still elusive and needs further investigation.

Thus, in this study, we show evidence of enhanced *in vitro* IL-27 signaling in T1D patient cells. Elevated IL-27Ralpha expression on the surface of mDCs, increased STAT3 phosphorylation and preeminent expression of inhibitory receptors after IL-27 stimulation suggest compensatory mechanisms due to ongoing inflammation and immunomodulatory role of IL-27 in T1D pathogenesis; however, the precise function of IL-27 *in vivo* needs further investigation.

## Patients and Methods

### Patients

A cohort of 51 pediatric patients with T1D (62.3% female) and 44 healthy donors (52.4% female) was included in this study. The median age of patients with T1D was 15.4 ± 2.3 years (range: 9.6–18.6 years), and the median age of the healthy donors was 18.5 ± 3.0 years (range: 12.2–21.6 years). All patients with T1D had been treated with insulin since disease onset. The median T1D duration was 5.9 ± 3.3 years (range: 1.2–15.3 years). The median of the last glycated hemoglobin (HbA1c) was 64 ± 14 mmol/mol (range: 39–94 mmol/mol). At the time of blood sampling, patients were metabolically stable; none of them had signs of active infection, neoplasia or other comorbidities except well-controlled celiac disease or autoimmune thyroiditis (5% of recruited patients with T1D). The healthy donors had a negative personal history of autoimmune diseases. Moreover, we included a cohort of T2D patients to the study. The median age of the patients was 54.8 ± 11.3 years (range: 42.6–75.9 years) with median duration of the disease 4.9 ± 5.8 years (range: 1.57–18.7). The median of glycated hemoglobin was 46.5 ± 8 mmol/mol. Not all patients were involved in all experiments due to the limited amount of blood available per sample.

Written informed consent was obtained from all the patients or the patients’ parents/guardians in accordance with the Declaration of Helsinki, and the study was approved by the Ethics Committee of University Hospital Motol.

### Cell isolation and culture

Peripheral blood was collected from patients and healthy volunteers into EDTA-coated tubes. Peripheral blood mononuclear cells (PBMCs) were isolated using Ficoll-Paque (GE Healthcare Biosciences, Uppsala, Sweden). The obtained cells were resuspended in RPMI 1640 medium with a sodium bicarbonate buffer system supplemented with 2% autologous serum, 1% penicillin and streptomycin and 1% Glutamax (ThermoFisher Scientific, Waltham, USA).

### Plasmacytoid and myeloid DC purification

PBMCs isolated from 20 T1D patients and 10 healthy controls were stained with FITC-conjugated antibodies against lineage-specific markers (CD3 clone MEM-57, CD19 clone LT19, CD20 clone LT20, CD16 clone LNK16, and CD56 clone MEM-188), anti-CD14-PEDy594 (clone MEM-15), anti-CD11c-APC (clone BU15) (Exbio, Prague, Czech Republic), anti-CD123-PE (clone 6H6) (ThermoFisher Scientific), and anti-HLA-DR-PC7 (clone L243) (BD Biosciences, Franklin Lakes, USA), washed, filtrated through 0.2 µm pore size and sorted under low pressure to increase viability and prevent activation on a FACSAria II (BD Biosciences). Myeloid DCs were gated as Lin-HLA-DR + CD14-CD11c+ cells, and plasmacytoid DCs were defined as Lin-HLA-DR + CD14-CD123+ cells. Sorted cells were recollected in tubes containing complete RPMI.

### Gene arrays

RNA was isolated from sorted mDCs and pDCs using the RNeasy Micro Kit following the manufacturer’s instructions (Qiagen, MD, USA). RNA concentration was measured with a spectrophotometer (Nanodrop), and RNA integrity was assessed by an Agilent 2100 bioanalyzer (Agilent, Santa Clara, USA). Total RNA was amplified (aRNA) using an Amino AllyI MessageAmp II aRNA Amplification Kit (Applied Biosystems, Foster City, USA). The RNA was then stained with Alexa 555 (Molecular Probes, Eugene, USA) and hybridized to a SurePrint G3 Human Gene Expression 8 × 60 K v2 Microarray (Agilent) according to the manufacturer’s instructions. The microarray was then scanned using InnoScan 900 (Innopsys, Carbonne, France). Microarray data processing and statistical analysis of differential gene expression were performed using the lima package in the R statistical program. Two-color microarray data analysis was performed according to the manufacturer’s recommendations. For each chip, raw intensity data were corrected for background, normalized by intra-array loess normalization and subjected to intra-array quantile normalization.

### IL-27 and soluble IL-27Ralpha serum levels

The concentration of sIL-27Ralpha in T1D sera was determined by ELISA (Sigma Aldrich, St. Luis, USA) following the manufacturer’s instructions. IL-27 was determined by Luminex (ThermoFisher).

### PD-L1 expression

PBMCs were cultured in complete media with or without rhIL-27 (100 ng/ml) overnight at 37 °C. Then, the cells were washed and stained with FITC-conjugated antibodies against lineage-specific markers (CD3 clone MEM-57, CD19 clone LT19, CD20 clone LT20, CD16 clone LNK16, and CD56 clone MEM-188), anti-CD14-PEDy594 (clone MEM-15), anti-CD11c-PB (clone BU15) (Exbio), anti-CD123-PerCP-Cy5.5 (clone 7G3) (BD Biosciences), anti-HLA-DR-Alexa700 (clone L243), anti-PD-L1-PC7 (clone 29E.2A3), and anti-CD86-Alexa647 (clone IT2.2) (BioLegend, San Diego, USA). The samples were acquired on a FACSAria II (BD Biosciences), and data analysis was performed using FlowJo software (TreeStar). Next, the expression of *PD-L1* in PBMCs was analyzed by RT-PCR after 100 ng/ml IL-27 stimulation for 5 hours.

### Surface marker expression

PBMCs were cultured in complete media with or without rhIL-27 (100 ng/ml) overnight at 37 °C and following antibodies were used: lineage-specific markers conjugated with FITC (CD3 clone MEM-57, CD19 clone LT19, CD20 clone LT20, CD16 clone LNK16, and CD56 clone MEM-188), anti-CD14-PEDy594 (clone MEM-15), anti-CD3-Alexa700 (clone MEM-15), anti-CD11c-APC (clone BU15) anti-CD86-PE (clone BU63) (Exbio), anti-CD123-PC7 (clone 6H6), anti-PD1-BV421 (clone EH12.2H7), anti-PDL1-BV510 (clone 29E.2A3), anti-gp130-PE (clone 2E1B02) (BioLegend), anti-HLA-DR-PerCP (BD Biosciences), anti-IL27Ralpha-PE (clone 191106) and anti-ADAM17-PE (clone 111633) (R&D Systems, Minneapolis, USA), respectively. The samples were acquired on a FACSFortessa (BD Biosciences), and data analysis was performed using FlowJo software (TreeStar).

### Phosphoflow

Whole blood was stimulated with 100 ng/ml rhIL-27 (Peprotech, New York, USA) for 15 minutes, 10 ng/ml rhIL-6, 500 ng/ml rhIFNα (both from R&D Systems) when indicated or left untreated at 37 °C. The optimal stimulation time was estimated after analyzing the effects of 5, 15 and 30 minutes of rhIL-27 stimulation on 8 healthy controls (Suppl. Fig. 2A). Intracellular signaling was prevented by using 4% paraformaldehyde without methanol (Sigma Aldrich) for 10 minutes at room temperature. Erythrocytes were lysed using 0, 1% Triton-X for 20 minutes (Sigma Aldrich) at 37 °C, leukocytes were permeabilized with ice-cold 80% methanol for 30 minutes and stained with a FITC-conjugated lineage antibody cocktail (CD3 clone MEM-57, CD19 clone LT19, CD20 clone LT20, CD16 clone LNK16, and CD56 clone MEM-188), anti-CD14-PEDy594 (clone MEM-15) (Exbio), anti-HLA-DR-Alexa700 (clone L243), anti-CD123-PC7 (clone 6H6) (BioLegend), anti-CD11c-APC (clone BU15) (Exbio), anti-phosphoSTAT1-BV421 (Tyr701) (clone 4a) and anti-phosphoSTAT3-PE (Tyr705) (clone 4/5-STAT3) (both from BD Bioscience). The samples were acquired on FACSAria II (BD Biosciences), and data analysis was performed using FlowJo (TreeStar).

### Western blot

PBMCs were stimulated for 15, 30 and 45 minutes with rhIL-27 (100 ng/ml) or left untreated. The cells were then washed and lysed with RIPA lysis buffer and PMSF (Cell Signaling, Danvers, USA), placed on ice, sonicated and then centrifuged at 14000 g to remove cell debris. For western blot analysis, samples were resuspended in Laemmli buffer (Sigma Aldrich) at a 1:1 ratio and boiled for 5 min. Proteins were separated by SDS-PAGE and transferred to PVDF membranes. After blocking with 5% BSA in TBST (TBS and 0.1% Tween, both from Bio-Rad, Hercules, USA), the membranes were incubated with the following primary antibodies: anti-STAT1 (clone EPYR2154) (Abcam), anti-phosphoSTAT1 (Tyr701) (clone M135), anti-STAT3 (clone D3Z2G), anti-phosphoSTAT3 (Tyr705) (clone D3A7) and anti-β-actin (clone D6A8) (all from Cell Signaling) overnight, followed by incubation with peroxidase-conjugated anti-rabbit or anti-mouse secondary antibodies for 2 hours. Membranes were developed using SuperSignal West Femto (ThermoFisher Scientific). Densitometry was performed with ImageJ software (National Institutes of Health, USA). Protein band area values were used for semiquantification. Graphs represent the ratio of stimulated/unstimulated cells of the band area calculated from the band area of phosphorylated forms/band area of unphosphorylated forms.

### RT-PCR

PBMCs were stimulated for 5 hours with rhIL-27 (100 ng/ml) or left untreated. RNA isolation, reverse transcription and RT-PCR were performed according to a previously published protocol^35^. Total RNA was isolated using the RNeasy Mini Kit following the manufacturer’s instructions (Qiagen, MD, USA), and complementary DNA (cDNA) was synthetized using M-MLV Reverse Transcriptase (ThermoFisher Scientific). RT-PCR was performed in duplicate using the obtained cDNA and Platinum Taq polymerase (ThermoFisher Scientific), 200 nM dNTP (Promega, Southampton, UK), 50 mM MgCl_2_ (ThermoFisher Scientific) and TaqMan primer/probe sets (ThermoFisher Scientific). Samples were matched to a standard curve generated by amplifying serially diluted products using the same PCR and normalized to *GAPDH* (forward primer GAAGGTGAAGGTCGGAGTC; reverse primer GAAGATGGTGATGGGATTTC; FAM/TAMRA CAAGCTTCCCGTTCTCAGCC) (TIB MOLBIOL, Berlin, Germany) to obtain the relative expression value. Real-time assays were run on an FX96 Cycler (Bio-Rad). The following primer/probe sets were used: *IL-27Ralpha* (Hs00956025_m1), *CXCL10* (Hs00171042_m1), *SOCS3* (Hs02330328_s1), *PD-L1* (Hs01125301_m1), *PD-L2* (Hs00228836_m1), *PD-1* (Hs00169472_m1), all from ThermoFisher Scientific.

### Statistics

The results were obtained from at least six independent experiments. Not all patients were involved in all experiments due to the limited amount of blood available per sample. Statistical analysis was performed using non-parametric one-way analysis of variance (ANOVA) with multiple comparisons Dunn’s post-test where applicable. A two-tailed paired Wilcoxon or unpaired Mann-Whitney *t*-test was also applied for data analysis using GraphPad Prism 8. Values of p < 0.05 (*), p < 0.01 (**) p < 0.001 (***) and p < 0.0001 (****) were considered statistically significant.

## Supplementary information

Supplementary Information
