# Supplementary material for: Enhanced STAT3 phosphorylation and PD-L1 expression in myeloid dendritic cells indicate impaired IL-27Ralpha signaling in type 1 diabetes
Source: Sci Rep. 2020 Jan 16;10:493. doi: 10.1038/s41598-020-57507-8 (PMC6965661; doi:10.1038/s41598-020-57507-8)

Enhanced STAT3 phosphorylation and PD-L1 expression in myeloid dendritic cells indicate impaired IL-27Ralpha signaling in type 1 diabetes

Z. Parackova[1*], P. Vrabcova[1], I. Zentsova[1], J. Kayserova[1], I. Richtrova[2], L. Sojka[1,2], K. Stechova[3], Z. Sumnik[4], A. Sediva[1]

[1]Department of Immunology, 2nd Faculty of Medicine, Charles University and Motol University Hospital, Prague, Czech Republic
[2]Sotio, a. s., Prague, Czech Republic
[3]Department of Internal Medicine, 2nd Faculty of Medicine, Charles University and Motol University Hospital, Prague, Czech Republic
[4]Department of Pediatrics, 2nd Faculty of Medicine, Charles University and Motol University Hospital, Prague, Czech Republic

***Correspondence:**
Zuzana Paračková, Department of Immunology, 2nd Faculty of Medicine Charles University, Faculty Hospital in Motol, V Uvalu 84, Prague 5, 15006, +420224435971, zuzana.parackova@fnmotol.cz

Supplementary Figure 1: Expression of (**A**) gp130, (**B**) ADAM17 on mDC surface and (**C**) IL-27Ralpha on T cell surface analyzed by flow cytometry. **(D)** Gating strategy for the detection of pSTAT1 and pSTAT3 in myeloid and plasmacytoid DCs. Statistical analysis was performed using Mann-Whitney unpaired *t*-test. Values of $p<0.05$ (*) were considered statistically significant.

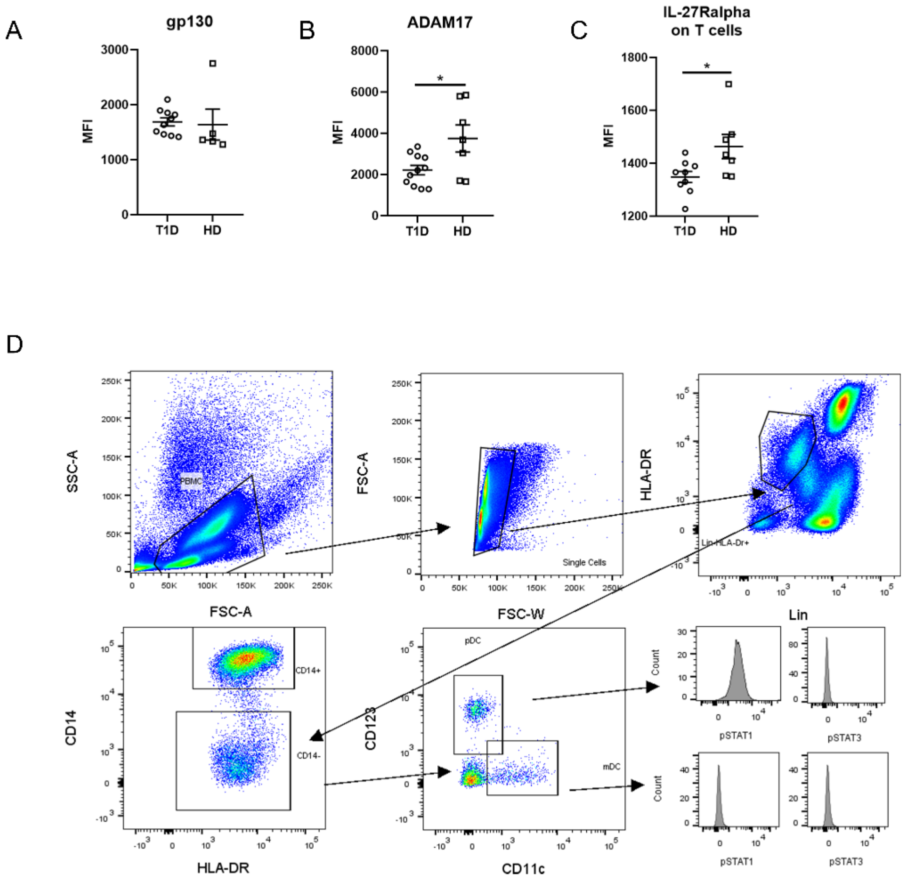

Supplementary Figure 2: STAT1 (Tyr701) and STAT3 (Tyr705) phosphorylation. (**A**). Time of stimulation optimization for phosphoflow cytometry experiments (n=8). (**B**) STAT1 and STAT3 phosphorylation detected by western blot in the PBMC compartment of T1D patients (n=6) and healthy donors (HD, n=3) after stimulation with rhIL-27 (100 ng/ml) for 15, 30 and 45 minutes. Graphs represent the ratio of stimulated/unstimulated cells of the protein band area calculated from the band area of phosphorylated forms/band area of unphosphorylated forms. (**C**) pSTAT1 and pSTAT3 detected by phosphoflow after whole blood from T1D patients (n=11) and HD (n=11) was exposed to rhIL-6 (10 ng/ml) for 5 minutes, rhIFNα (500 ng/ml) for 15 minutes or left untreated, respectively. Statistical analysis was performed using the Kruskal-Wallis test with Dunn's multiple comparisons the Wilcoxon paired or Mann-Whitney unpaired *t*-test. Values of p<0.05 (*), p<0.01 (**), p<0.001 (***) and p<0.0001 (****) were considered statistically significant.

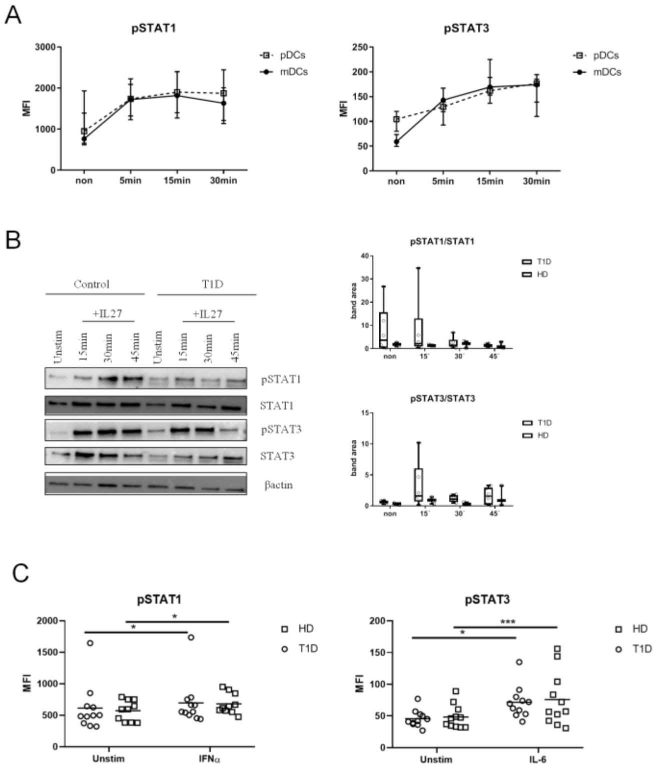

Supplementary Figure 3: Uncropped gels. (**A**) pSTAT1, (**B**) STAT1, (**C**) pSTAT3, (**D**) pSTAT1 (**E**) β actin

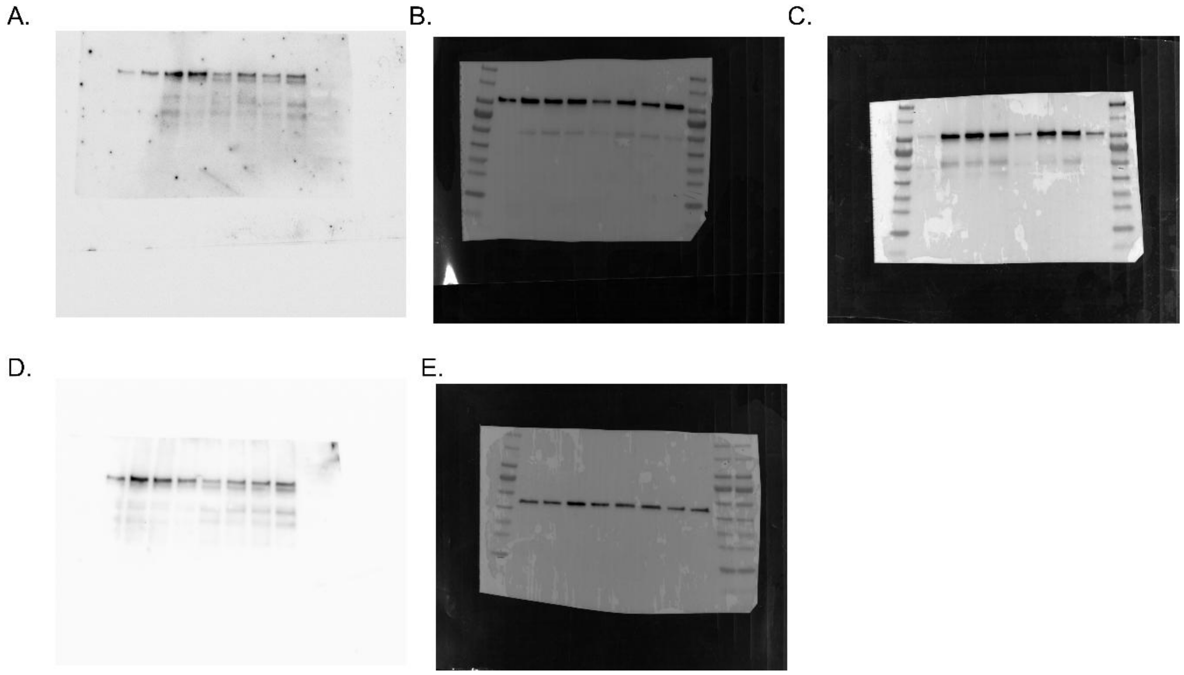

Supplementary Figure 4: Type 2 diabetes and IL-27 signaling. (**A**) IL-27Ralpha expression on myeloid dendritic cells (mDCs) surface analyzed by flow cytometry in 10 T2D patients, 7 HD and 9 T1D patients. (**B**) pSTAT1 (Tyr701) and pSTAT3 (Tyr705) detected in mDCs was analyzed by phosphoflow after whole blood from T2D patients (n=8), and healthy donors (HD, n=10) was stimulated with 100 ng/ml rhIL-27 for 15 minutes. Values are expressed as MFI (mean fluorescence intensity). (**C**) PD-L1 and CD86 expression on mDC surface detected after overnight incubation of PBMCs from T2D patients (n=7) and healthy donors (n=8) with rhIL-27 (100 ng/ml) analyzed by flow cytometry. Statistical analysis was performed using the Kruskal-Wallis test with Dunn's multiple comparisons. Values of p<0.05 (*), p<0.01 (**), p<0.001 (***) and p<0.0001 (****) were considered statistically significant.

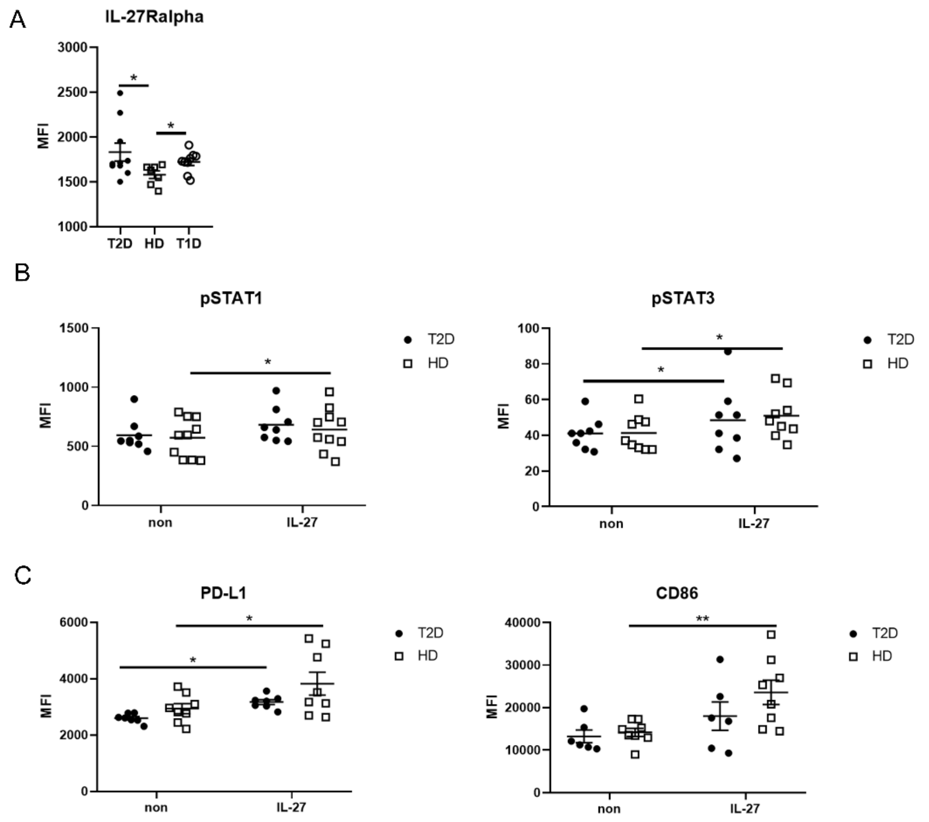

Supplement: Supplementary file 1 — Supplementary Information [file 41598_2020_57507_MOESM1_ESM.pdf]
